# Clustered local transmission and asymptomatic *Plasmodium falciparum *and *Plasmodium vivax malaria *infections in a recently emerged, hypoendemic Peruvian Amazon community

**DOI:** 10.1186/1475-2875-4-27

**Published:** 2005-06-23

**Authors:** OraLee Branch, W Martin Casapia, Dionicia V Gamboa, Jean N Hernandez, Freddy F Alava, Norma Roncal, Eugenia Alvarez, Enrique J Perez, Eduardo Gotuzzo

**Affiliations:** 1 Department of Medicine, Geographic Medicine, University of Alabama at Birmingham, Bevill Research Building BBRB-556, Birmingham, Alabama, 35294-2170, USA; 2 Direccion de Salud-Loreto, Ministerio de Salud (MINSA), Iquitos, Peru; 3 Instituto de Medicina Tropical "Alexander Von Humboldt", Universidad Peruana Cayetano Heredia, A.P. 4314 Lima 100, Lima, Peru

## Abstract

**Background:**

There is a low incidence of malaria in Iquitos, Peru, suburbs detected by passive case-detection. This low incidence might be attributable to infections clustered in some households/regions and/or undetected asymptomatic infections.

**Methods:**

Passive case-detection (PCD) during the malaria season (February-July) and an active case-detection (ACD) community-wide survey (March) surveyed 1,907 persons. Each month, April-July, 100-metre at-risk zones were defined by location of *Plasmodium falciparum *infections in the previous month. Longitudinal ACD and PCD (ACP+PCD) occurred within at-risk zones, where 137 houses (573 persons) were randomly selected as sentinels, each with one month of weekly active sampling. Entomological captures were conducted in the sentinel houses.

**Results:**

The PCD incidence was 0.03 *P. falciparum *and 0.22 *Plasmodium vivax *infections/person/malaria-season. However, the ACD+PCD prevalence was 0.13 and 0.39, respectively. One explanation for this 4.33 and 1.77-fold increase, respectively, was infection clustering within at-risk zones and contiguous households. Clustering makes PCD, generalized to the entire population, artificially low. Another attributable-factor was that only 41% and 24% of the *P. falciparum *and *P. vivax *infections were associated with fever and 80% of the asymptomatic infections had low-density or absent parasitaemias the following week. After accounting for asymptomatic infections, a 2.6-fold increase in ACD+PCD versus PCD was attributable to clustered transmission in at-risk zones.

**Conclusion:**

Even in low transmission, there are frequent highly-clustered asymptomatic infections, making PCD an inadequate measure of incidence. These findings support a strategy of concentrating ACD and insecticide campaigns in houses adjacent to houses were malaria was detected one month prior.

## Introduction

**Figure 1:**
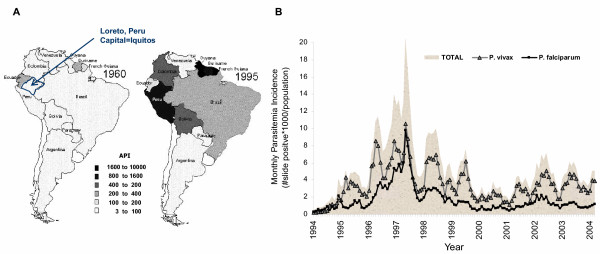
**A-B: History of malaria in Peru. **Before 1960, there was *P. vivax*, *P. malariae *and a very limited number of *P. falciparum *cases. After DDT campaigns stopped in the 1960's, malaria went from a low annual parasitaemia incidence (**API **= 1000*slide positive/population), limited to the northern costal regions, to a high level in 1995 (**A**: directly from Roberts *et al*[1], with permission). In 1991 *P. vivax *reemerged and in 1994 *P. falciparum *emerged in Loreto. An epidemic ensued, focused near the capital city of Loreto, Iquitos, and a hypoendemic continues (**B**).

In Peru, the history and prevalence of the human-malaria causing *Plasmodium *species parasites are different from what is found in the widely studied African, Asian or Pacific countries. The incidence of malaria in Peru before 1940 is not well-known, but between 1940 and 1960 there were reports of *Plasmodium vivax *and *Plasmodium malariae *infections in northern and central Peru. In the 1960's malaria transmission was very low due to an impressive eradication attempt. However, in the 1980's the eradication campaign was abandoned and house spraying programs were increasingly neglected. *Plasmodium falciparum *was first reported in Peru's Department of Loreto in 1988 [1]. Then, between 1991 and 1995, the annual infection prevalence dramatically increased in the coastal regions near Piura [2] (Figure 1A). In the region surrounding the capital city of Iquitos, there was a *P. vivax *and *P. falciparum *epidemic between 1995 and 1998 (Figure 1B). This is attributable to the abandonment of DDT campaigns leading to the increased geographic range or increased local abundance of the mosquito vector *Anopheles darlingi *[2,3].

Since 1998, in Loreto, malaria has continued to be detected in low incidence, with the *P. falciparum *and *P. vivax *annual infection incidence varying between 5 and 50 cases/1000 persons (Figure 1B). Malaria infections are recorded by the Peruvian Ministry of Health, Ministerio de Salud Dirección de Salud Loreto (MINSA). The health system is highly centralized, with little access to private physicians even within the city of Iquitos [4]. The primary approach to malaria control is passive case detection in local MINSA health posts or centers/hospitals and administration of effective anti-malaria drug treatment at no cost to the patient [4]. This control method is in line with Pan American Health Organization (PAHO) program guidelines. Based upon this detection and treatment method, the malaria incidence is exceedingly low, but has been sustained since the emergence of *P. falciparum *and *P. vivax *10 years ago. However, passive case detection might not accurately reflect the malaria transmission rate or be an effective malaria intervention strategy if there are local transmission clusters and asymptomatic infections are going undetected.

One potential explanation for the sustained low incidence of malaria is a clustering of infections into a small subgroup of the population. If this occurred, then one would expect that the actual infection frequency in some persons would be higher than that estimated by summarizing over the entire population.

Of particular interest is whether the malaria transmission is occurring within the suburban villages just outside of Iquitos, where MINSA regularly applies pyrethoid insecticides to houses [4], or whether infections are acquired through travel to more remote regions. Because passive case detection is summarized at the community level, by number of infections detected per number of individuals living within the community, the more local malaria transmission dynamics needed to target insecticide campaigns remain unresolved. Insecticide spraying must be better targeted, as the insecticides are expensive, short-lived and are rapidly washed away from the resin-rich houses during the frequent rains in this region [4]. If the transmission was occurring in remote regions, or if transmission was clustered within certain locations within the suburban villages, then insecticide campaigns could be targeted to these infection-prone regions.

Also, the occurrence of local transmission impacts the expectation of having asymptomatic infections. If the majority of infections occur within a sub-population who frequently travel or live in higher transmission regions, then these highly exposed individuals might receive the quantity of infections conventionally considered necessary develop protective immunity against symptoms during infection [5-17]. In this case, these individuals would likely have infections undetected by passive case detection. Conversely, if infections are acquired locally, then there would be low transmission in the whole population and one would expect most of the infections to be symptomatic. This expectation is based upon literature regarding the development of naturally acquired immunity to *P. falciparum *malaria requiring frequent infections over a period of 5 years and sustained frequent infections in order to maintain an ability to resist symptoms during malaria infection [5-17].

Having to conduct active case detection in order to detect asymptomatic infections would not be expected in a region with recent and low transmission. Data suggests that, early in the epidemic, symptoms were likely, although deaths were rare. From the MINSA records, Ambarru et al. (1998) calculated the malaria-attributable death rate in 1997 to be only 0.1% [1]. This low mortality during the epidemic of 1995–1998 might be partly attributable to under-reporting of mortality. Another possible explanation of low morbidity and mortality with *P. falciparum *infections is good health care. It is possible that people experience symptoms quickly and go to the health centers immediately. If this is the case, then passive case detection with administration of effective malaria treatment drugs at no cost to the patients is an effective intervention strategy which can explain both the low mortality and the failure of malaria transmission to increase in this region.

The frequency of asymptomatic malaria infection is not known in this or most low transmission regions. There has been a longitudinal study in Brazil which demonstrated asymptomatic *P. falciparum *and *P. vivax *malaria infections [18]. In Peru, studies have been limited by cross-sectional designs. At the end of the epidemic (1998) in one community just north of Iquitos, there was a comprehensive cross-sectional active and passive case detection study [19]. Roper et. al (2000) found that 90% of both *P. falciparum *infections and *P. vivax *infections detected were associated with symptoms and the parasite densities were similar to that expected in non-immune individuals [19]. However, in 1999, Roshanravan et al conducted a cross-sectional survey of 998 individuals living in communities south of Iquitos [20]. They found 13 individuals with *P. falciparum *and 30 with *P. vivax *by microscopy. Of these, only 8 and 19, respectively, reported a fever. The conclusion was that there were many low density malaria infections that were asymptomatic. However, the question remains whether these were early stage infections that would have subsequently produced symptoms and then have been detected at the health post.

Understanding the continued low malaria transmission and the potential for increased transmission in the Amazon Jungle region of Peru is contingent upon determining the prevalence of a capable malaria vector, clustering of infections within certain households and the presence of asymptomatic malaria. This present study, in a suburban community near Iquitos, determined the level of local malaria transmission, the frequency of individuals presenting with symptoms and the dynamics of *P. falciparum *and *P. vivax *infections during weekly active surveillance. The results suggest that, even in low transmission, active case detection provides critical insight into *P. falciparum *and *P. vivax *transmission and infection and that active case detection can be spatially and temporally targeted for a feasible malaria intervention strategy.

## Methods

### Study area

South of Iquitos, the San Juan District, is a focus of *P. falciparum *and *P. vivax *malaria transmission. The rural communities in San Juan have *P. falciparum *and *P. vivax *infections, mostly detected in the rainy season, which lasts from January to July. Typically, MINSA personnel detects, treats and documents malaria cases by passive-case detection. All persons presenting with a fever at the community-based MINSA health posts or a larger health clinic located in the urban region of San Juan are tested for malaria by microscopy. Individuals go to the MINSA health centers as there is no access to private doctors and anti-malaria treatment is given by MINSA at no cost to the patient. The anti-malaria drug supply is tightly controlled and given as observed therapy (see treatment schedule, below) each day to those infected.

**Figure 2:**
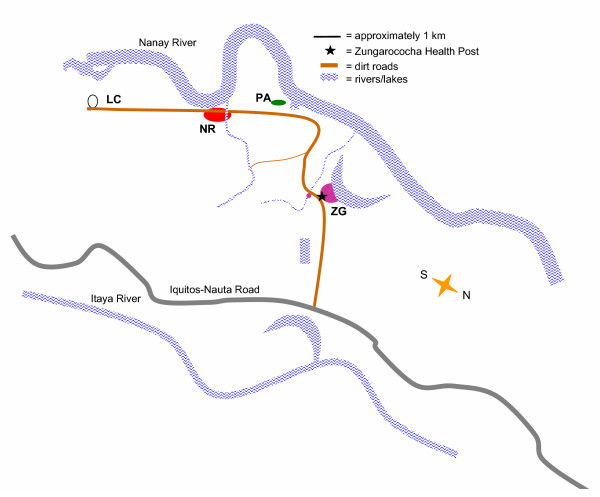
**Schema of community in the passive and active surveillance study. **Zungarococha is a suburban community approximately 5 kilometres (km) from Iquitos, Peru. Zungarococha is composed of four villages: Zungarococha town (ZG, N = 1293), Puerto Almendra (PA, N = 207), Ninarumi (NR, N = 472), and Llanchama (LL, N = 142). Passive case detection occurred in the MINSA health post. Active case detection first included a community survey in March, 2003. Then, from the *P. falciparum *infections detected in passive case detection or the community survey, at-risk regions were defined for each month April-July, 2003, based upon the location of the month prior's *P. falciparum *infections. The location of these *P. falciparum *infections were used to determine a 100-metre radius at-risk zone for the following month. In each month of April-July 2003, 27–39 sentinel houses (approximately 150 participants/month) were selected within each at-risk zone for one month of weekly prospective visits.

The community in which this study was conducted, Zungarococha, was selected based upon the prevalence of *P. falciparum *malaria infections, the acceptance by the community and the fact that the community is composed of four villages each separated by approximately two kilometres and yet serviced by the same MINSA health post. Zungarococha (population N = 1907) is composed of 4 villages: Zungarococha town (ZG), Puerto Almendra (PA), Ninarumi (NR) and Llanchama (LL) (Figure 2). The road to LL was not consistently passable by a four-wheel drive truck, so it was not included in the active house-by-house follow-up. The environment and income level in these communities is similar. The village "ZG" is more developed and is the location of the MINSA health post. There is a bus that offers daily transportation between villages. All of the villages are primarily sustained by agriculture and fishing. Generally, women work in or near their homes. Men often work in agriculture or are construction/maintenance workers at the agricultural university or on commercial farms within the community. Fishing is also conducted on the rivers which border the villages. In each village, the houses are typically made of wood with resin-rich thatch roofs, although approximately 10% of the homes are cement-block. There are no screens on houses, and mosquitoes have easy entry. There is electricity in some homes and streets of ZG and PA. The homes are on average 8 × 12 metres in size and are often side-by-side. In most cases the houses are close together in lines that follow dirt streets. More than 70% of the houses are less than 6 metres apart. The homes are often located near the marshy areas of rivers or ponds. Generally, the houses face dirt streets while the back of the houses are open to more foliage-dense areas within 100 metres of rivers or ponds. Much of the cooking and many other activities occur in these "back-yard" riparian habitats. The exception to this village design is NR. In NR, half of the village is traditional with many homes close together, while the other half is more dispersed.

### Entomology

Members of the community have been informed that *An. darlingi *mosquito is highly prevalent in their community and causes malaria transmission. Public health education is conducted both by local MINSA health promoters and radio announcements. Additionally, throughout the daily interactions with the community and village authorities, the MIGIA investigator team discussed how to decrease the possibility of *An. darlingi *biting. All of the households included in the survey answered a questionnaire which included questions on bed net and insecticide usage. More than 95% of the households reported using bed nets, although none of the households reported using insecticide treated bed nets or repellants in their homes. Approximately twice per year, the MINSA entomologists conduct fumigation campaigns and apply pyrethoid insecticides to all houses [4].

Entomology capture studies were conducted to determine the transmission potential within the Zungarococha villages. Mosquitoes were captured by mechanical aspiration from 7–12 pm, the known time of peak biting for the anthropophilic *An. darlingi *mosquito malaria vector in the Peruvian Amazon [4]. *An. darlingi *lives near and bites within human households (endophagic behavior). *An. darlingi *also bites near its breeding sites, which are typically shady edges of quiet rivers (riparian habitats) [21]. Each month, April-July, 2003, mosquitoes were collected on five porches each week for one month one the porches of sentinel houses (different houses each month). These houses were sentinel houses selected within the defined at-risk zones for a given month (see below), with five houses per at-risk zone enrolled each month for weekly entomological captures. Each entomology team (composed of two members) placed the captured mosquitoes in containers labeled by location and hour of collection. The mosquitoes were identified to the species level by expert MINSA entomologists by morphology while using a dissecting microscope. *An. darlingi *have an easily distinguishable one white front leg.

### Passive case detection

This study had a full-time clinical team stationed at the Zungarococha Health Post for passive case detection throughout the year. The MINSA records of malaria detection within the Zungarococha Health Post were reviewed and complied in order to calculate the *P. vivax *and *P. falciparum *incidence. The number of malaria cases per given time were divided by the total population (N = 1,907) to determine the incidence by passive case detection. The records were matched by name to the individuals observed in active case detection (see below).

### Active case detection

Active case detection was conducted during the months of March and July, 2003 (the malaria transmission season).

The active case detection began with a cross-sectional community-wide survey, wherein all family members were invited for malaria parasite testing and a health survey. Between March 31 and April 5, 2003, there were village-wide surveys of Zungarococha. For simplicity within this manuscript, the community survey is stated to have occurred in March, 2003.

From May until July, 2003, longitudinal active case detection included a monthly selection of houses (selected at random, or as close to random as possible for field conditions, see below) located within 100 metres of an "index" house. Houses were called "index" houses if they had at least one person detected with a *P. falciparum *infection in the previous month, detected through the community survey results, the health post detection, or the prior month's active case detection. An approximate 100 metre radius circle around the index houses (or a median point between index houses when there were two index houses spaced by less than 50 metres) was defined as an at-risk zone for *P. falciparum *transmission. If at-risk zones overlapped in a given month, houses were assigned to one of the zones. Each month, all houses within each 100 metre at-risk zone were identified by assigning house numbers (addresses) on a map of each village and entered as list of possible sentinel houses. Of all possible sentinel houses (generally, 20–50 potential houses existed within a given 100 metre at-risk zone), 10 potential sentinel houses from each at-risk zone were randomly selected. In June and July, this selection was done by a random number generator (conducted using Statistical Analysis Software Version 8, Cary, NC). The enrolment team for this study would be given this list and then would go to these potential sentinel houses one at a time to recruit all household members for one month of weekly active sampling until at least 30 individuals (living in 5–9 houses) were enrolled within each at-risk zone. The index house were included in this longitudinal active case detection, however, the results from active case detection are not included in this analysis due to these houses not being selected at random.

There were 5–6 at-risk zones during each month of active surveillance, with approximately 150 sentinel individuals enrolled each month, April-July, 2003. Each household member was visited 4 times (once per week for one month), unless there was a positive blood slide that resulted in extra visits. The recruitment and retention was high: 82% of the time all permanent members of each sentinel house consented and enrolled and 86% of consenting individuals completed participation in all 4 weekly visits.

For each month of active weekly surveillance, due to the relatively small size of each village (less than 600 square-metres) and the location of index cases, approximately the same zones were defined in successive months of active surveillance. Throughout the study, 70–90% of the houses within PA and NR, and 50–70% of the houses within ZG were included within the zones of our active surveillance.

### Epidemiological questionnaire, physical and blood samples

At each visit, whether in the health post, the community visits or the weekly home-visits, a detailed epidemiological questionnaire was administered and a physical examination by a physician was conducted. Axillary temperature was measured by a digital thermometre. 0.25–3 ml of blood, by fingerprick or venipuncture, was collected. Individuals diagnosed with malaria had 3–6 ml of blood collected by venipuncture. Thin and thick blood smears and capillary haematocrit tubes were prepared. The remaining blood was stored in tubes with EDTA, separated by centrifugation, and the sera and packed blood cells were frozen within 18 hours. Haematocrit was measured as packed cell volume (PCV) after centrifugation.

### Microscopy

There were two microscopists in this study, each having more than 15 years experience. Their ability to for accurate reading of blood slides is well known, and they are the designated quality control monitors for all MINSA malaria microscopy in the Department of Loreto. Blood smears were stained with Giemsa using standard procedure. Using 100× magnification to read the thick smear, all malaria species' trophozoites and gametocytes were counted separately. Microscopy fields were read to count at least 500 white blood cells (WBCs) before diagnosing an individual as negative by microscopy. The parasite density for each species (parasites/μl blood) was determined by number of parasite species (trophozoites and gametocytes counted separately) multiplied by 6,000 and then divided by the total number of WBCs counted. Using 6,000 RBCs per one WBC to determine parasite density was established by the MINSA microscopists after years of counting RBCs and WBCs in malaria infected patients from this region and their continued intermittent checks verify that this remains an appropriate average conversion factor.

### Treatment

In passive case detection, where individuals presented at the Zungarococha Health Post, treatment was administered immediately upon diagnosis within the Health Post. In active case detection, household visits occurring in the afternoon, the blood slides were transported to the microscopists' laboratory for reading either on the same day or the following morning. Blood slides from individuals reporting febrile illness within the previous 2 days, having a detected fever ≥ 38.3°C, or having a haematocrit PCV <30% were flagged for immediate reading by microscopists. Blood slides from individuals not reporting the symptoms listed above were read within 6 days after the actual visit and collection of the blood slide. All individuals were revisited on "day i+7." If individuals found positive from the earlier blood slide were now symptomatic at this "day i+7" visit, they were given antimalaria drug treatment. If they remained asymptomatic, the blood smear was flagged for immediate reading. These participants were then re-visited in one day ("day i+8") for a confirmation slide and treatment of all individuals with a positive blood slide on "day i" or "day i+7". Even without symptoms, all cases were treated within 7 days of confirmation of malaria parasites. If a positive slide was detected at the last of the 4 weekly visits, then an extra visit, including an extra blood sample, was made. The physician-nurse team was in the vicinity throughout the study to take a blood smear from anyone presenting with malaria symptoms, even if they were not in the regularly scheduled weekly visits. Again, symptomatic individuals had their blood slides read within one day.

Whether in active or passive case detection, all treatments were given through the MINSA authorities, following the MINSA National Drug Policy Guidelines. *P. vivax *treatment is chloroquine (10 mg/kg for 3 days) with primaquine (0.5 mg/kg for 7 days). *P. falciparum *treatment is mefloquine (12.5 mg/kg daily for 2 days) with artesunate (4 mg/kg daily for 3 days) in non-pregnant patients older than 1 year of age. In pregnant women and infants, *P. vivax *is treated with chloroquine (10 mg/kg daily for 2 days and 5 mg/kg on third day) and *P. falciparum *is treated with clindomyacin (10 mg/kg 2 times daily for 5 days) and quinine (10 mg/kg 3 times daily for 7 days). The treatments are specific to *Plasmodium *species, as *P. falciparum *parasites from this region are highly resistant to chloroquine[22]. The treatments are effective at eliminating all stages of the *Plasmodium species *parasites to which they are directed. In all cases the treatments are observed, and a fingerprick blood sample is collected at 7 days and then again at 14 days later to confirm treatment success.

### Data analysis

Data was entered using a programme in ACCESS developed specifically for this cohort project. The principle investigator developed a program that displays potential matching records by similarity (keyed to name, address, and age). The principle investigator and data management specialist determined when records from the health post matched individuals in our active follow-up. After the matching, all names and unnecessary information was removed from the analysis data set. Relational database management and statistical analysis was conducted using the Statistical Analysis Software Version 8 (SAS, Cary, NC). The Fisher's Exact Test (Fisher's exact) was used to compare the expected versus observed frequency of detecting infections in houses and to compare the frequency of febrile illness associated with *P. falciparum *versus *P. vivax *infections. Parasite densities were logarithmically transformed (base 10) in order to normalize the densities and calculate the geometric mean and 95% confidence interval (95%CI). A general linear model logistic regression (GLM logistic) was used to test the correlation between probability of febrile illness and parasite density, while controlling for age.

### Human subjects ethical approval

All protocols were reviewed and approved by the University of Alabama at Birmingham, Universidad Peruana Cayetano Heredia and the Peruvian Ministerio de Salud (MINSA) Institutional Review Boards for the study of human subjects. Written, informed consent was obtained from all participants. In the case of minors less than seven years old, the parents or guardians gave consent. In the case of minors between seven and eighteen, both assent from the minor and consent from the parents or guardians was obtained prior to enrollment.

## Results

### Entomology

**Figure 3:**
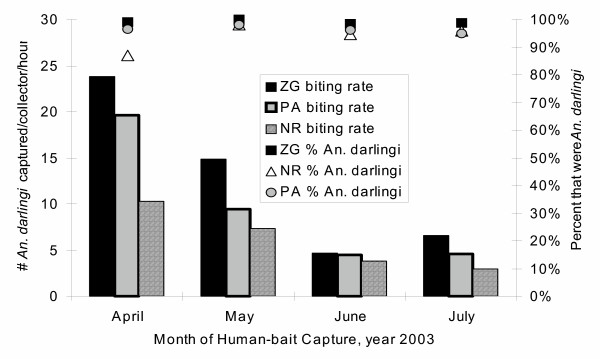
Mean *An. darlingi *captured, prepared to bite/person/hour and percent of mosquitoes that were *An. darlingi*. Captures were conducted by mechanical aspiration from 7–11 pm (the known peak biting time), on the porch of households4. *An. darlingi *are known to bite in and near human households[4]. There were 10 MINSA entomologists, working in pairs, to give a total of 5 entomologic captures/night. The households were chosen from the sentinel houses in at-risk zones selected for human infection surveillance. In at-risk zones where there were more than 5 houses, 5 of the sentinel houses were randomly selected for entomologic captures. Each month, 5 at-risk zones were included, with 5 houses each, for 4 weekly captures in each house.

*Anopheles darlingi *is an anthropophilic mosquito that bites in human houses as well as in the riparian habitats[21] between the backs of houses and the quiet rivers which are within each of the Zungarococha villages. Between April, 2003 and July, 2003, 12,035 mosquitoes were captured, of which 87% were *An. darlingi *(Figure 3). These human-bait captures (by mechanical aspiration) were on the porches of houses within our at-risk zones for *Plasmodium falciparum *transmission, so defined by having a *P. falciparum *infection centrally located within the 100-metre radius circle one month prior. In total, there were 80 houses, each sampled 4 times (weekly), giving 160 entomological captures between the months of April and July, 2003. In April, the month with the highest *An. darlingi *biting rate, the mean household biting rate was 10–24 *An. darlingi *bites/person/hour in each village. The biting rate rapidly decreased in the months of May-July, 2003, leading to the dry months of August-December (data not shown). This data supports the previous MINSA reports that there is seasonal *P. falciparum and Plasmodium vivax *transmission by *An. darlingi*[4] and demonstrates high local transmission potential.

### Incidence of infection by passive case detection

In the health post, there were 60 *P. falciparum *and 539 *P. vivax *infections detected by passive case detection between January, 2003 and March, 2004 (one was a mixed-species infection). Only 11 *P. falciparum *and 121 *P. vivax *infections were detected during the low transmission months of January, 2003 and August-December, 2003. Considering infections detected February-July, 2003 and estimating by dividing the total infections detected by the total population size (1907), the passive-case detection incidence was 0.03 *P. falciparum *and 0.22 *P. vivax *infections/person/malaria season (Table 1). This is significantly lower than prevalence of infection detected in individuals living in at-risk zones for *P. falciparum *transmission.

**Table 1:** Comparison of *P. falciparum *and *P. vivax *passive and active case detection. The study includes individuals followed throughout the year-2003 malaria transmission season (February-July, 2003). Passive case detection was conducted at the Health Post, where individuals presenting with febrile illness are tested for malaria parasites by microscopy. Active case detection was performed in two ways. In March, a community survey was conducted where 957 of the population (N = 1907) were sampled. Then, April-July, sentinel individuals living in houses defined as at-risk zones for *P. falciparum *transmission were randomly selected. Individuals selected as sentinels were followed prospectively for one month with weekly visits. The passive and "passive and active" (active+passive) prevalence only considers individuals who were enrolled both in the community survey and enrolled as sentinels in at-risk zones for at least one month during these months.

	***P. falciparum***	***P. vivax***
**Total malaria cases detected by passive case detection **February - July, 2003 / total population	49/1907 = 0.03	418/1907 = 0.22

No. monthly active case intervals with weekly sampling in at-risk zones (No. of individuals)	592 (573)	592 (573)

**Malaria infections detected by active+passive surveillance in at-risk zone sentinels**	Total: 74/573 = 0.13	Total: 224/573 = 0.39
Passive case detection, February – July	15^a ^(20.8%)	48 (21.4%)
Community survey, March	12^b ^(16.7%)	36^b ^(16.1%)
Monthly active surveillance in at-risk zones, April – July	46 (62.5%)	140 (62.5%)

**Difference in passive versus active+passive prevalence **Attributable fraction potentially explained by sampling in at-risk zones and/or asymptomatic infections	0.13/0.03 = 4.33	0.39/0.22 = 1.77

^a ^Of the remaining 34 *P. falciparum *infections detected in passive case detection, but not included in this count, at least 12 occurred in individuals living within at-zones. This is because these individuals were not selected as sentinels for the weekly active surveillance.

^b ^Five additional *P. falciparum *and 36 additional *P. vivax *infections were detected in the community survey. However, they occurred in individuals not selected as sentinels for the weekly active surveillance.

### Prevalence of infection by active case detection

There were 957 participants in the cross-sectional survey in March. Then, based upon the detection of *P. falciparum *infections in the community survey and Health Post, 100-metre radius circles within each village were defined, focused from a house where there was a *P. falciparum *infection detected the month prior. Each month, approximately 150 individuals living in 5–6 at-risk zones were selected as sentinels. There were a total of 593 instances of weekly active surveillance for one month between April and July, 2003. Only 19 individuals were enrolled for more than one month of active sampling. In total, 74 *P. falciparum *and 224 *P. vivax *infections were detected by microscopy (3 were mixed *P. falciparum *and *P. vivax *infections). Of these infections, 45 *P. falciparum *and 140 *P. vivax *infections were detected during the one month of weekly active case surveillance of randomly selected sentinel individuals living in at-risk zones.

The age and sex distribution of all 573 individuals enrolled as sentinels in the at-risk zones was investigated (Table 2). Gender was not associated with prevalence of infection. Children (0–12 years-of-age) had a lower prevalence of infection than older individuals, which was statistically significant (p < 0.0001 for both *P. falciparum *and *P. vivax *infection) (Table 2). From the background knowledge of the community, this increase in prevalence is attributable to young children more frequently being in beds, under bednets, during the peak *An. darlingi *biting time of 7–12 pm.

**Table 2:** Age and sex distribution of individuals enrolled for active surveillance in at-risk zones. Age groups were classified so as to group children and adolescents separately from adults, while maintaining at least 15 individuals in each age group for each infection status. The prevalence of infection was significantly different by age group, where the children had less *P. falciparum *and *P. vivax *infections (Fisher's Exact Test, p < 0.0001). There was no significant difference in the frequency of males in the total population versus the *P. falciparum *or *P. vivax *infected individuals as a whole or when stratifying by age group (Fisher's Exact Test, p > 0.2).

age group (years)	Enrolled in at-risk zone for weekly sampling		*P. falciparum*		*P. vivax*		active+passive prevalence	
			
	n =	% Male	N =	% Male	N =	% Male	*P. falciparum*	*P. vivax*
**0–11**	214	49%	19	53%	48	52%	0.09	0.22
**12–20**	119	41%	19	47%	62	51%	0.16	0.52
**21–104**	240	46%	36	39%	114	48%	0.15	0.48

**Total**	**573**	**46%**	**74**	**45%**	**224**	**50%**	**0.13**	**0.39**

^a ^Of the remaining 34 *P. falciparum *infections detected in passive case detection, but not included in this count, at least 12 occurred in individuals living within at-zones. This is because these individuals were not selected as sentinels for the weekly active surveillance. ^b ^Five additional *P. falciparum *and 36 additional *P. vivax *infections were detected in the community survey. However, they occurred in individuals not selected as sentinels for the weekly active surveillance.

### Comparing passive and active case detection

The passive and active (active+passive) case detection prevalence was calculated from all individuals who had passive case detection in the health post, the community survey and active-weekly detection for at least one month. The active+passive prevalence was 0.13 *P. falciparum *and 0.39 *P. vivax *infections/person/malaria season (Table 1).

To compare the active+passive to the passive case detection a ratio was computed (Table 1). Because individuals in active case detection were randomly selected within each at-risk zone and had the same follow-up throughout the study, there was no obvious bias to the prevalence calculated within at-risk zones. Likewise, because there was a community survey, community-wide advisories to go to the Health Post upon experiencing malaria symptoms, and accessibility to free malaria treatment at the Health Post, there was no obvious bias to the incidence calculated from passive case detection in the whole community. The *P. falciparum *and *P. vivax *active+passive case detection was 4.33-times and 1.77-times higher, respectively, than the prevalence predicted by passive case detection in the health post (Table 1).

The greater increase in active+passive versus passive case detection prevalence for *P. falciparum *in comparison to *P. vivax *was expected. This is because the active case detection was focused in regions defined as at-risk for *P. falciparum *based upon the previous months' *P. falciparum *infections. Additionally, to explain the 4.33-times higher prevalence when including active-case detection in at-risk zones, the symptoms and dynamics of the infections was investigated. However, first, the analysis regarding the further clustering of infections within at-risk zones is presented.

### Spatial and temporal clustering of infections within at-risk zones

**Figure 4:**
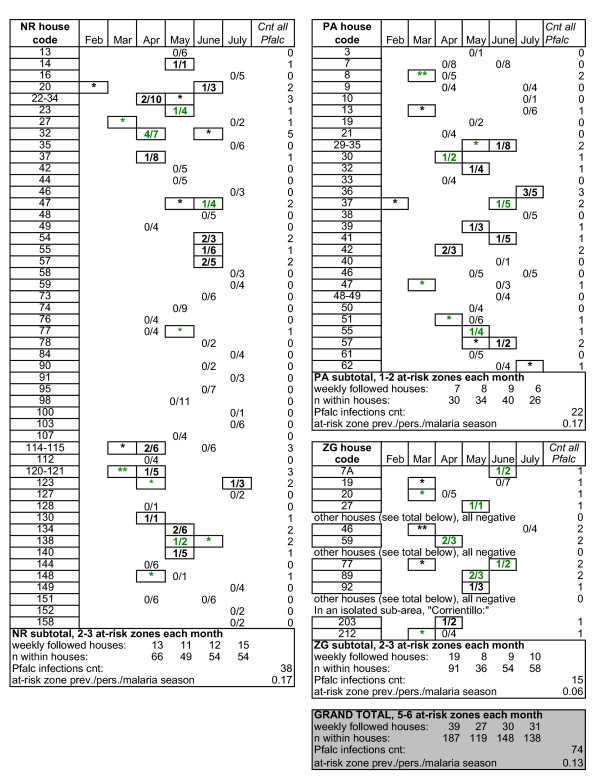
**Spatial and temporal clustering of *P. falciparum *(Pfalc) infections within at-risk zones**. Pfalc detection prompted our selection of sentinel houses within at-risk zones in the following month. There was clustering of infections (outlined in black) in the same and adjacent houses. The houses followed a general linear arrangement, with consecutive numbers being contiguous houses. Generally, the house numbers wrap around in opposite order for opposing houses located across a street. Human subjects concern prohibits display of more precise house locations. All persons with active case detection are shown (except for ZG, where, for simplicity and space constraints, the many households that were negative for Pfalc are not listed). Asterisks are passive case detections in the Health Post occurring within the sentinel houses. The index houses defining at-risk zones for the following month are shown in green font. Active sampling events are shown as a ratio. The numbers of Pfalc infected individuals detected in the household are shown as the numerator. The numbers of persons in the house participating as sentinels are shown as the denominator. There were four instances of persons living in two houses: where members were sampled within one of the two houses and reported frequently eating diner and sleeping in both houses (these are shown as dashed-house codes).

There were *P. falciparum *and *P. vivax *infections in each village, with *P. falciparum *being more prevalent in NR and PA and *P. vivax *being more prevalent in ZG (Figure 4). The plateau of *P. falciparum *prevalence suggests that local transmission was decreasing after May, 2003. This is consistent with the entomologic results showing significantly low *An. darlingi *biting May-July, 2003.

A short-term clustering of infection within homes is expected if there is local entomologic transmission. There were many instances of detecting more than one *P. falciparum *or *P. vivax *infection within a given household (Figure 4). Considering the number of individuals living in each house, there was a significant clustering of infections within adjacent houses. The expected frequency of detecting a given number infections is the number of individuals living in households with the given infection history multiplied by the probability of the occurrence. For *P. falciparum *and *P. vivax*, separately, the expected frequency of having one, two, three or more infections was calculated and compared to the observed frequency. In each case, there was a significant difference between the expected and observed frequencies of having more than one infection in a given household (Fisher's Exact Test, p < 0.0001 for each species). Even more striking was the clustering when considering adjacent houses (Figure 4).

Of those surveyed over time, 3 individuals had more than one *P. falciparum *infection and 39 individuals had more than one *P. vivax *infection, separated by at least one month after malaria drug treatment, during the February-July, 2003 active+passive surveillance. Due to the malaria treatment protocol being effective at clearing all stages of the malaria parasites and the negative blood smears observed after the treatment, these following infections were termed "discrete." Detecting more than one discrete *P. vivax *infection within a given individual was more common. In fact, 4 persons had 3 discrete *P. vivax *infections. The 74 *P. falciparum *infections were detected in 69 individuals (4% of the individuals had more than one *P. falciparum *infection). The 212 *P. vivax *infections were detected in 173 individuals (23% of the individuals had more than one *P. vivax *infection). Detecting an individual with more than one infection for a given species was not different from the frequency expected based upon the active+passive prevalence (probability of having more than one infection is 0.13^2 ^for *P. falciparum *and 0.39^2 ^for *P. vivax*) (Fisher's Exact, *P. falciparum *p = 0.6197, *P. vivax *p = 0.4354).

The results show low-level local transmission even within at-risk zones, with the same and adjacent houses being at high risk in the following month (Figure 4). Even if there is long-term clustering of infections in the same households over successive seasons, the expected infection rate would still be less than 0.5 *P. falciparum *and less than 1.5 *P. vivax *infections/person/year. For example, of the 573 individuals in our at-risk zone active surveillance, there were 110 who lived in houses where more than one person within the house had a *P. falciparum *infection during the 2003 malaria season. The prevalence in this subpopulation within the at-risk zones is only 0.26 *P. falciparum *infections/person/malaria season. Therefore, even in the population at high *P. falciparum *infection risk, the transmission would be defined as hypoendemic based upon infection prevalence by standards based upon African transmission[5].

### Travel away from homes

Participants were asked if they traveled away from their village for their occupations or other reasons during the hours of 6 pm-12 pm. Twenty-nine participants reported travel to a rural location or travel to any location by river within one month of active case detection. Of these 29 travelers, there were 7 (24%) *P. vivax *and 4 (14%) *P. falciparum *infections detected within one month of the time of travel. Therefore, this travel to rural regions with assumed higher malaria transmission (MINSA reports) did have a higher frequency of malaria infection. However, the other 58 *P. falciparum *and 205 *P. vivax *infections detected in active case detection were in 544 individuals not reporting travel to more rural regions. Therefore, the majority of infections detected in Zungarococha (94% for *P. falciparum *and 97% for *P. vivax*) could not be explained by travel away from their villages.

### Description of infections detected in active surveillance

In the active case detection (community survey and weekly surveillance), 62 *P. falciparum *and 212 *P. vivax *infections were detected. The geometric mean parasite density, considering the first positive blood smear obtained in active surveillance, was 1282 *P. falciparum *parasites/μl blood (95%CI: 1201–1369) and 321 *P. vivax *parasites/μl blood (95%CI: 312–331). Of particular interest regarding the transmission potential, 53% of the *P. falciparum *and 22% of the *P. vivax *infections had gametocytes detected. The geometric mean gametocyte density was 151 *P. falciparum *gametocytes/μl blood (95%CI: 134–171) and 120 *P. vivax *gametocytes/μl blood. The higher gametocytemia frequency of *P. falciparum *relative to *P. vivax *is attributable to the difficulty in distinguishing *P. vivax *gametocytes from trophozoites by microscopy.

A classic indicator of clinical malaria is the presence of a fever at the time parasites are detected. The frequency of a measured fever in individuals with parasites in blood smears was 21% for *P. falciparum *cases and 14% for *P. vivax *cases, respectively (Fisher's exact: p = 0.2329). The symptoms reported in individuals with *P. falciparum *or *P. vivax *infections at any time within one month were considered. Of the 62 individuals with *P. falciparum *infections detected in active case detection, 41%, 3%, 6%, 6%, 5% and 6% reported fever, chills, headache, diarrhea, nausea/vomiting or body aches, respectively. Of the 212 individuals with discrete *P. vivax *infections detected in active case detection, 24%, 1%, 4%, 9%, 5% and 10% reported fever, chills, headache, diarrhea, nausea/vomiting or body aches, respectively. The proportion of individuals with a detected or reported fever (febrile illness) was significantly higher in *P. falciparum *versus *P. vivax *infections (Fisher's exact: p = 0.0096). There were no signs of severe malaria infections (hyperparasitaemia >100,000/μl, neurological symptoms, severe anemia, or respiratory distress) during active surveillance. One *P. vivax *infection was associated with jaundice.

The geometric mean parasite density of *P. falciparum *infections in the group with febrile illness was 4011/μl (95%CI: 811–9746). In individuals with no reported or detected fever at anytime within one month of the detected *P. falciparum *parasitaemia, the geometric mean parasite density was 973/μl (95%CI: 142–2078). The parasite density of *P. vivax *infections in the group with febrile illness was 636/μl (95%CI: 81–2477) versus 391/μl (95%CI: 173–1211) in the group without febrile illness. For *P. falciparum*, there was a positive correlation between probability of febrile illness and *P. falciparum *parasite density and (GLM logistic: p = 0.0203), independent of the negative correlation between probability of febrile illness and age (GLM logistic: p = 0.0471). *P. vivax *parasite density was not significantly correlated with probability of febrile illness and (GLM logistic: p = 0.0833).

For asymptomatic individuals, enrolled in weekly surveillance, blood smear microscopy results were not available and reported to the participant until the next scheduled visit, seven days later. This protocol was necessary to prioritize microscopy reading on symptomatic participants and to maintain a logical schedule of participant follow-up visits. Of the 45 individuals in the weekly follow-up visits who had *P. falciparum *infections, 30 individuals were asymptomatic with no indication of parasites at the time of sampling. However, upon reading their blood smears by microscopy, parasites were detected. The initial day of parasitaemia detection was termed "day i." The follow-up visit of these "day i" positive but asymptomatic individuals was termed "day i+7." At this regularly scheduled visit of these 30 individuals, the regular blood smear was collected and read by microscopy on the same day. However, sixteen individuals did not have parasitaemia upon the "day i+7" visit and remained asymptomatic. Six of the sixteen individuals were treated, even though they were negative by microscopy at the time of treatment, "day i+7". Ten of the sixteen individuals with earlier transient parasitaemias were not given treatment, opting for continued surveillance on the following day ("day i+8"). All ten of these asymptomatic individuals with transient parasitaemias on "day i", but negative on "day i+7" remained asymptomatic and were again negative "day i+8", and despite continued surveillance throughout the study remained without parasitaemia.

In the 14 cases where asymptomatic parasitaemias were not self-limited to being undetectable by microscopy, the parasite densities were often decreased in the "day i+7" blood sample taken just before treatment. From the 30 asymptomatic individuals, the geometric mean parasite density in the "day i" visits was 1047 parasites/μl blood (95%CI: 951–1153). Considering the 14 individuals with parasitaemia on "day i+7" the geometric mean parasite density was 905 parasites/μl blood (95%CI: 758–1082). The parasite density was higher in the "day i+7" versus the "day i" blood smear in only 9 of the 30 (20%) of the asymptomatic individuals. Therefore, it appeared that the majority of asymptomatic individuals were self-limiting their infections and would not have been detected with malaria if it were not for active case detection.

## Discussion

*Plasmodium falciparum *and *Plasmodium vivax *malaria have been continuing in low transmission, as detected by passive case detection, since the 1995–1998 epidemic. This study determined the presence of a competent malaria vector, the local spatial and temporal clustering of infections and the prevalence of both symptomatic and asymptomatic parasitaemia in a suburban community.

The potential for local transmission by *Anopheles darlingi *was observed in each village of Zungarococha. Local transmission was demonstrated by finding that over 90% of the infections were in individuals not reporting travel outside of the village. The *An. darlingi *biting rate, especially in April, 2003, was high (10–24 *An. darlingi *bites/person/hour, with an estimated 5 hours of *An. darlingi *biting per night). *Anopheles darlingi *is the most competent malaria vector in South America. However, *An. darlingi *appears to be a less competent vector for malaria transmission than the *Anopheles species *prevalent in Africa[23]. Malaria studies in Africa, where the *P. falciparum *prevalence is higher, generally report that 1–20% of the important vector species are infected with *P. falciparum *sporozoites[24]. The high *An. darlingi *biting rate and the high proportion of human infections with gametocytes (53% of the *P. falciparum *infections) suggests ample potential for malaria transmission within each village.

The infection incidence by passive case detection in the Zungarococha Health Post was compared to the infection prevalence by active and passive case detection (active+passive) in 100-metre zones defined as at-risk to local *P. falciparum *transmission, based upon the detection of a *P. falciparum *infection within the preceding 30 days. The Health Post passive detection showed a population incidence of 0.03 *P. falciparum *and 0.22 *P. vivax *infections/person/malaria season. In individuals living in at-risk zones, participating in the cross-sectional survey and participating in weekly visits for one month as randomly selected as sentinels, the active+passive case detection prevalence was 0.13 *P. falciparum *and 0.39 *P. vivax *infections/person/malaria season. Therefore, the *P. falciparum *prevalence detected by active+passive case detection in at-risk zones was 4.77-fold higher than the passive case detection.

One attributable-factor for the 4.77-fold increase in detecting *P. falciparum *infections within at-risk zones versus the overall population prevalence is the clustering of infections within at-risk zones in Zungrococha. Passive case detection summarizes the infection incidence by considering the number of infections per the population size of the community. However, because infections were clustered in certain regions of the community, using the whole population as the denominator underestimated the prevalence. A household clustering of malaria infections was detected in a study by Brooker et al[25]. Brooker et al determined that households where children were low weight, households in lower altitudes and household where there were not drugs kept in the house were more likely to have *P. falciparum *infections[25]. In our cohort, the socioeconomic status, environment, house construction, and access to drugs (highly regulated by MINSA) are all very homogenous. Given the homogeneous environment, the same-house and adjacent-house clustering is most likely attributable to mosquito behavior: that is, to highly localized mosquito biting. The results are consistent with a concept that although *An. darlingi *has the potential to fly one kilometre or more to find a blood meal, *An. darlingi *and other *Anopheles *species frequently remain in highly restricted localities where hosts are readily available[4,21,26]. The clustering of infections suggests that *An. darlingi *return for multiple feedings in the same or adjacent houses. Future genetic typing of the malaria parasites both in the human host and mosquito vector will compare the genetic relatedness of the spatially (adjacent houses) and temporally (within one month) clustered infections versus the infections detected in other regions within the villages.

Another attributable-factor for the increased rate of detecting malaria infections within at-risk zones, as compared to the detection in the overall population, is the detection of asymptomatic infections. This study used a broad definition of symptoms: including any fever (reported or observed) or anemia (haematocrit <20% PCV) during the entire month of active follow-up. The symptoms reported from individuals detected with *P. falciparum *or *P. vivax *infections were relatively mild. There was a correlation between febrile illness and *P. falciparum *parasite density, which was independent of the correlation between febrile illness and age, as has been detected in other studies[27]. However, even with a wide range of parasite densities observed in this study, only 41% and 24% had a febrile illness within one month of the detected *P. falciparum *or *P. vivax *infection, respectively. This frequency of febrile illness is lower than that reported by a cross-sectional study in this region conducted by Roshanravan et al, in 1999, only one year after the epidemic's onset[20]. In the new study, presented here, the longitudinal dynamics of parasitaemia in asymptomatic individuals were investigated. In 80% of the asymptomatic cases, the parasitaemia decreased by the end of the one-week sampling interval (the one-week follow-up visit). Of the asymptomatic *P. falciparum *infections, 53% (16/30) had no detectable parasitaemia when they were re-visited or on successive checks for parasitaemia during the following 7 days. Moreover, 10 of the 16 individuals had continued surveillance by at least one additional blood sample 14 days later and continued surveillance by passive case detection throughout the malaria season. Thus, all available evidence suggested that these infections were self-limited.

Having infection follow-up more frequently than 3 times in one week (as in this study) has occurred in a few studies of individuals who had many prior malaria infections. Daily sampling for over two weeks on semi-immune individuals with asymptomatic *P. falciparum *infections in Papua New Guinea, Senegal and Tanzania showed that *P. falciparum *parasites remained detectable by microscopy in almost all blood samples [12-15]. As in the studies of semi-immune individuals described above [12-15], the parasite densities of asymptomatic individuals in this study were, by definition, ≥5,000 parasite/μl. Despite the similar parasite densities under consideration, in this low transmission Peruvian community, 16 of the 30 asymptomatic individuals (53%) had no detectable parasitemia in the subsequent follow-up visits. A precise understanding of self-limited infections will require further study of more individuals.

Considering the frequency of asymptomatic infections, with symptoms observed for one month, the results of this study suggest that approximately 40% of infections detected by active case detection would not have been detected by passive case detection in the Health Post. Asymptomatic malaria infections have also been detected in a longitudinal study in Brazil, where transmission was higher than this Peruvian cohort but still considered low transmission [18]. Of particular importance in this Peruvian population study, the prevalence of the sexual stage gametocytes was considered. There were asymptomatic cases with circulating gametocytes, providing the potential to infect mosquitoes and cause additional human cases. Therefore, although against the conventional thought that *P. falciparum *protective immunity requires years of frequent *P. falciparum *infections to develop [5-17], the need for active case detection in low transmission regions must be considered.

This study compared the malaria incidence by passive case detection for the total community with the prevalence rates in the 100-metre at risk zones for malaria. The limitation of not having an *a priori*-defined monthly control group in non-at-risk zones is that the incidence of asymptomatic malaria infections within these non-at-risk zones could not be calculated. Now that the possibility of asymptomatic infections in this community is known, a future study will include such control groups for conducting cost-benefit analysis.

The results of this present study, however, are not significantly limited by lacking precise knowledge of asymptomatic infections in non-at-risk zones. First, the attributable-fraction of the increased prevalence in at-risk zones can be estimated by assuming that the frequency of asymptomatic infections in at-risk zones was similar to that in non-at-risk zones. Supposing that, without active case detection, 40% of the *P. falciparum *infections would have gone undetected in the health post, then there is an approximately a 2.6-fold increase (4.33*0.60 = 2.6) in the *P. falciparum *active+passive prevalence versus the passive population incidence attributable to clustering of infections within at-risk zones. Second, the spatial and temporal clustering of infections was apparent within at-risk zones, with adjacent houses being at highest risk within at-risk zones. By focusing resources for active case detection in the at-risk zones, more than three times the malaria infections were detected and treated versus passive case detection. Future studies will determine the range of focused active case detection and fumigation campaigns to obtain the most cost-effective method for immediate public health and long-term decreases in malaria transmission.

## Conclusion

The occurrence of clustered infections in at-risk zones and asymptomatic infections contributed to the higher prevalence by active case detection within at-risk zones versus passive case detection generalized to the community population. Currently, passive case detection and treatment is the primary approach to malaria control in Peru. This control method is consistent with PAHO's program guidelines, which do not advocate active case detection. However, according to this longitudinal study in a low transmission community, this control strategy only targets approximately one-third of the malaria infections. Therefore, in regions where active case detection cannot be sustained throughout a community, feasible alternatives to detect and limit malaria transmission are greatly needed.

Carter et al. discussed the importance of concentrating often limited and expensive resources and manpower where they would be most effective [26]. Studying spatial dynamics of malaria infection in low transmission regions might be a particularly effective model system for determining how to target malaria control efforts [26]. Continued investigation in this community will determine if there is a long-term clustering of infections in houses across malaria transmission seasons. At that point, a retrospective definition of malaria risk at the household level, using mapping aided by Global Positioning Satellite technology, can be used to better target control measures. Although more investigation and cost-benefit analysis is needed, the study presented here suggests one more immediate and readily applicable intervention strategy.

It is evident that passive case detection, even in low transmission, does not identify the regions most at risk for infection and has the potential of leaving many asymptomatic malaria cases undetected. However, community-wide active case detection in regions of low transmission is highly cost prohibitive for local governments. Additionally, by focusing in regions more at risk to transmission, community participation in active case detection is more easily sustained. The results of the present study suggest a specific public health strategy where resources for active case detection are limited: *after detecting a malaria infection in a house in a given month by passive case detection, in the following month actively survey for malaria infections in this and adjacent houses and focus insecticide spraying in these at-risk houses*.

## Authors' contributions

OHB designed the project, supervised the field team and directed the research team. OHB performed the data management, analysis and writing of the manuscript. WMC coordinated collaboration with MINSA and provided critical background statistics on malaria in Iquitos, such as Figure 1B. DG assisted in developing the consent forms and questionnaires and supervised the storage of samples and integration with the Peruvian University, UPCH-IMTavH. JNH, physician and coordinator, conducted physical examinations, asked epidemiologic and clinical questionnaires as well as coordinated efforts in the field. FFA was the lead field entomologist as well as the participant recruitment specialist responsible for selecting the sentinel houses each month for active case detection and obtaining and continually verifying participants' informed consent. NR organized the field team, kept daily follow-up records and maintained logistics between the clinic sampling and laboratory team. EA conducted daily field visits, acquiring the microscopy results, recording data and determining haematocrit packed cell volume from each visit. EP supervised the morphologic characterization of the mosquitoes into *species *and conducted the entomology analysis. EG coordinated the collaboration between UAB, the Peruvian University, UPCH-IMTavH, and MINSA. All authors have read and approved of the content of this manuscript.
